# Metabolic characteristics and prognostic differentiation of aggressive lymphoma using one-month post-CAR-T FDG PET/CT

**DOI:** 10.1186/s13045-022-01256-w

**Published:** 2022-03-26

**Authors:** William G. Breen, Matthew A. Hathcock, Jason R. Young, Roman O. Kowalchuk, Radhika Bansal, Arushi Khurana, N. Nora Bennani, Jonas Paludo, Jose C. Villasboas Bisneto, Yucai Wang, Stephen M. Ansell, Jennifer L. Peterson, Patrick B. Johnston, Scott C. Lester, Yi Lin

**Affiliations:** 1 Department of Radiation Oncology, Mayo Clinic, Rochester, MN USA; 2 Department of Biomedical Statistics and Informatics, Mayo Clinic, Rochester, MN USA; 3 Department of Radiology, Mayo Clinic, Rochester, MN USA; 4 Division of Hematology, Department of Medicine, Mayo Clinic, 200 First Street SW, Rochester, MN 55905 USA; 5 Department of Radiation Oncology, Mayo Clinic, Jacksonville, FL USA

**Keywords:** CAR-T, PET/CT, Non-Hodgkin lymphoma

## Abstract

**Background:**

F-18 fluorodeoxyglucose positron emission tomography computed tomography (PET/CT) is used to assess response of non-Hodgkin lymphoma (NHL) to chimeric antigen receptor T cell (CAR-T) therapy. We sought to describe metabolic and volumetric PET prognostic factors at one month post-CAR-T and identify which patients with partial response (PR) or stable disease (SD) are most likely to subsequently achieve complete response (CR), and which will develop progressive disease (PD) and death.

**Methods:**

Sixty-nine patients with NHL received axicabtagene ciloleucel CAR-T therapy. One-month post-CAR-T infusion and PET/CT scans were segmented with a fixed absolute SUV maximum (SUVMax) threshold of 2.5 using a semiautomated workflow with manual modification to exclude physiologic uptake as needed. Metabolic tumor volume (MTV), total lesion glycolysis (TLG), SUVMax, and other lesion characteristics were calculated and associated with risk of PD and death.

**Results:**

Patients with total MTV > 180 cc, presence of bone or parenchymal disease, SUVMax > 10, single lesion TLG > 245 g, or > 2 total lesions had increased risk of death. Patients with total MTV > 55 cc, total TLG > 250 cc, SUV Max > 10, or > 2 total lesions had increased risk of PD. For the subset of 28 patients with PR/SD, higher SUVMax was associated with increased risk of subsequent PD and death. While 86% of patients who had SUVMax ≥ 10 eventually had PD (HR 3.63, 1.13–11.66, *p* = 0.03), only 36% of those with SUVMax < 10 had PD.

**Conclusions:**

Higher SUVMax at one month post-CAR-T is associated with higher risk of PD and death. SUVMax ≥ 10 may be useful in guiding early salvage treatment decisions in patients with SD/PR at one month.

**Supplementary Information:**

The online version contains supplementary material available at 10.1186/s13045-022-01256-w.

Chimeric antigen receptor T cell (CAR-T) therapy provides an opportunity for long-term remission in patients with aggressive relapsed or refractory B cell non-Hodgkin lymphoma (NHL) [1–4]. However, the rate of disease progression after CAR-T therapy is still significant, and advancements in risk stratification are needed to further tailor therapy and monitoring.

In clinical practice, F-18 fluorodeoxyglucose (FDG) positron emission tomography computed tomography (PET/CT) is routinely obtained at baseline and then serially for response assessment and surveillance. Despite advancements in PET/CT imaging and analysis, current standards divide treatment response into only four categories: complete response (CR), partial response (PR), stable disease (SD), or relapsed/progressive disease (PD) [5]. Patients who have a CR to therapy have excellent prognosis with a fraction potentially cured, while those with PR or SD have a more mixed prognosis, with approximately half of patients eventually achieving CR [1, 2]. However, it is unknown which of these patients with PR/SD are more likely to convert to CR and can be safely monitored for continued response, and which should be considered for immediate salvage therapy to prevent PD that would limit further treatment options.

Metabolic PET/CT characteristics including metabolic tumor volume (MTV) and total lesion glycolysis (TLG) represent opportunities to provide more accurate prognosis and guide therapy, particularly for patients with SD or PR who have uncertain prognoses [6–9]. We sought to analyze metabolic and volumetric PET/CT prognostic factors at one month post-CAR-T and identify which patients with PR/SD are more likely to experience subsequent PD and death (complete methods in supplement).

Sixty-eight patients with NHL were treated with axi-cel CAR-T therapy between January 2018 and July 2020 (Additional file 1: Table S1). Of these, 27 (39%) achieved CR by Lugano criteria, 24 (35%) achieved PR, 4 (6%) had SD, and 13 (19%) experienced PD. With a median follow-up of 13.3 months (interquartile range 4.7–18.0 months), the OS at 6, 12, and 18 months was 75%, 65%, and 42%, respectively. EFS at 6, 12, and 18 months was 43%, 39%, and 37%, respectively. At last follow-up, 46 patients (67%) had experienced PD and 30 (43%) had died.

When analyzing one-month post-CAR-T infusion PET/CT for all 69 patients regardless of Lugano classification, multiple characteristics representing increased disease burden (MTV) and bulk (TLG of the largest lesion) were associated with increased risk of death and PD (Additional file 1: Tables S2 and S3).

Twenty-eight patients (41%) had either PR or SD at one-month post-CAR-T infusion and were analyzed separately for predictors of PD and death. Of these, 12 (43%) had experienced progression at last follow-up. SUVMax was significantly associated with risk of PD as a continuous variable and when using a cutoff point of 10 (HR 3.63, 95% CI 1.13–11.66, *p* = 0.03) (Fig. 1A). While 86% of patients with PR/SD who had SUVMax > 10 at one month post-CAR-T eventually had PD, only 36% of those with SUVMax ≤ 10 had PD (Fig. 1A, B). No other PET characteristic was significantly associated with risk of subsequent PD in patients with PR/SD one-month post-CAR-T infusion. For patients with PR/SD at one month, those with SUVMax ≥ 10 had high subsequent PD rates and should be monitored closely and considered for early salvage.

**Fig. 1:**
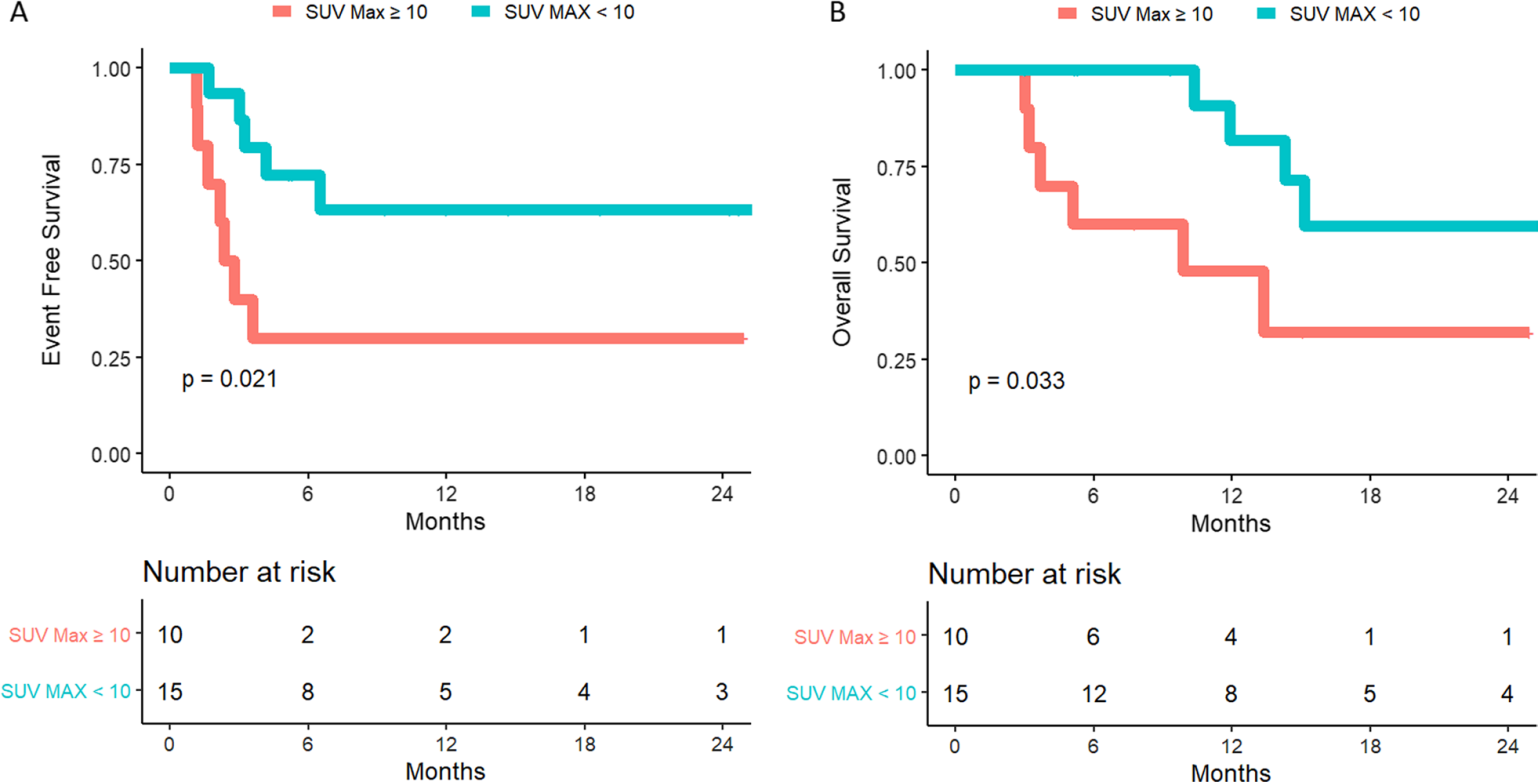
Risk of progression (**A**) and death (**B**) stratified by SUVMax > 10 for patients with PR/SD at one month post-CAR-T. Patients with SUVMax > 10 at one-month post-CAR-T infusion had higher risk of death and progression than patients with SUVMax < 10, as demonstrated by the Kaplan–Meier curves above

Currently, the Lugano criteria are still used for PET/CT assessment at one-month post-CAR-T infusion, dividing patients into only four broad categories, with significant clinical and prognostic heterogeneity between patients in the same category (Fig. 2A, B) [10]. Extracting and utilizing metabolic and volumetric data present in routinely obtained PET/CT scans may provide better prognostic discrimination and guidance of future treatments [7, 11, 12]. While several metabolic characteristics were analyzed, this study identified SUVMax ≥ 10 as a simple and effective prognostic predictor for patients with PR/SD at one-month post-CAR-T infusion. Using SUVMax > 10 to guide management one month after CAR-T infusion is a simple step beyond the Lugano system that may improve management decisions for this patient population. This is consistent with the recently published work from MD Anderson; combined, these two single institution studies support SUVMax > 10 as a useful biomarker one-month post-CAR-T infusion [6].

**Fig. 2:**
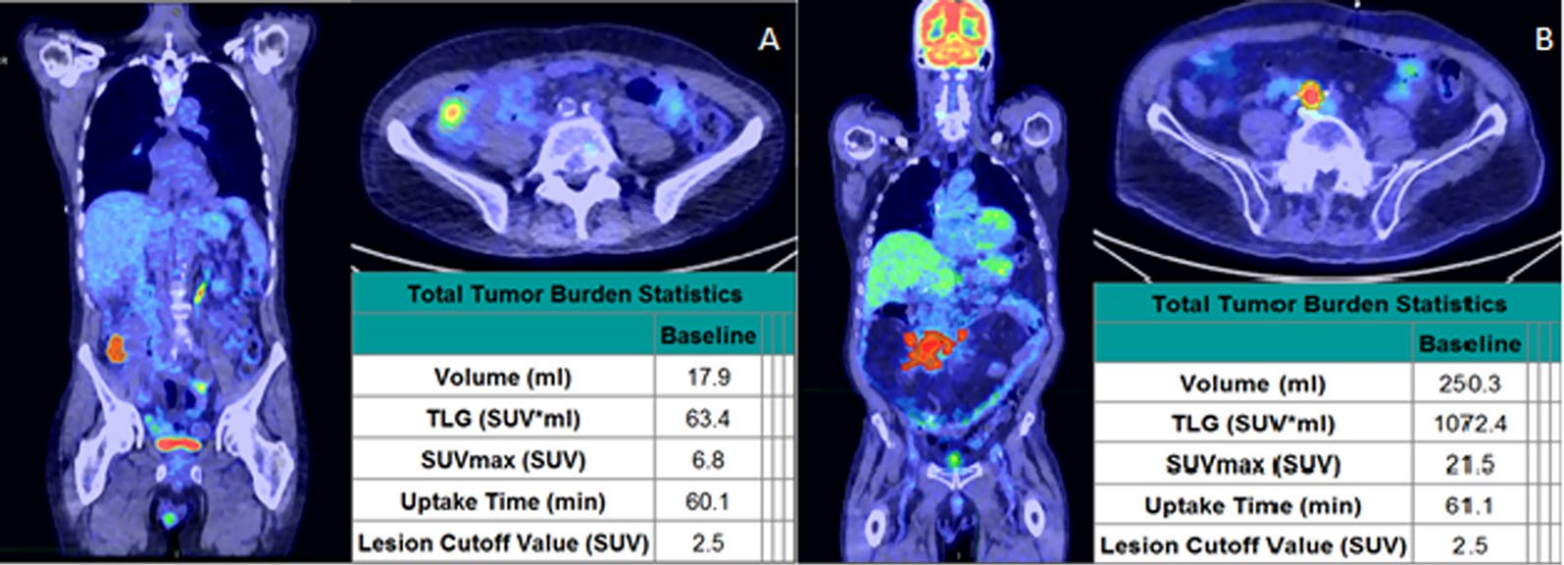
One-month post-CAR-T PET imaging and analysis. Two patients both assessed as having partial response one-month post-CAR-T therapy. The patient on the left pane (**A** SUVMax < 10) subsequently converted to complete response and is without progression at last follow-up, while the patient on the right (**B** SUVMax > 10) had subsequent progression and death

This analysis is limited by its retrospective nature, size, and the lack of widespread assessment of MTV and TLG in clinical practice. Fortunately, SUVMax ≥ 10 was identified as the key metric, which is readily evaluable on all PET/CT scans. Prospective validation of these findings is needed.

## Supplementary Information

**Additional file 1.** **Supplemental Tables. Supplemental Table 1:** Patient and Treatment Characteristics. **Supplemental Table 2:** Risk of Progressive Disease According to One Month Post-CAR-T Infusion PET/CT Characteristics for All Patients. **Supplemental Table 3:** One-Month Post-CAR-T Infusion PET/CT Characteristics and Risk of Death. **Supplemental Table 4:** Association between PET/CT Characteristics and Death in Patients with PR or SD One-Month after CAR-T.

## Data Availability

The datasets used and/or analyzed during the current study are available from the corresponding author on reasonable request.
