# Supplementary material for: Metabolic characteristics and prognostic differentiation of aggressive lymphoma using one-month post-CAR-T FDG PET/CT
Source: J Hematol Oncol. 2022 Mar 26;15:36. doi: 10.1186/s13045-022-01256-w (PMC8962609; doi:10.1186/s13045-022-01256-w)
Supplement: Supplementary file 1 — Additional file 1. Supplemental Tables. Supplemental Table 1: Patient and Treatment Characteristics. Supplemental Table 2: Risk of Progressive Disease According to One Month Post-CAR-T Infusion PET/CT Characteristics for All Patients. Supplemental Table 3: One-Month Post-CAR-T Infusion PET/CT Characteristics and Risk of Death. Supplemental Table 4: Association between PET/CT Characteristics and Death in Patients with PR or SD One-Month after CAR-T. [file 13045_2022_1256_MOESM1_ESM.docx]

| **Supplemental Table 1: Patient and Treatment Characteristics** | | |
| --- | --- | --- |
| **Patients** |  | **N=69 (100%)** |
| Age at CAR-T infusion | Median (years) | 61 (range 26-76) |
| Sex | Female | 26 (38%) |
| Disease Subtype | Diffuse Large B-Cell Lymphoma | 39 (57%) |
|  | Transformed Follicular Lymphoma | 16 (23%) |
|  | High-grade Lymphoma | 13 (19%) |
|  | Primary Mediastinal B-Cell Lymphoma | 1 (1%) |
| Previous Lines of Therapy | 2 | 14 (20%) |
|  | 3 | 29 (42%) |
|  | ≥4 | 26 (38%) |
| B Symptoms at CAR-T Evaluation | Present | 7 (10%) |
|  | Absent | 61 (88%) |
|  | Unknown | 1 (1%) |
| LDH Level at CAR-T Evaluation | Elevated | 19 (28%) |
|  | Normal | 48 (70%) |
|  | Not Evaluated | 2 (3%) |
| CRP at CAR-T Infusion | >100 mg/L | 6 (9%) |
|  | 10-100 mg/L | 42 (61%) |
|  | ≤10 mg/L | 21 (30%) |
| Ferritin at CAR-T Infusion | Elevated | 25 (36%) |
|  | Normal | 44 (64%) |
| Received Bridging Therapy | Yes | 44 (64%) |
|  | No | 25 (36%) |

| **Supplemental Table 2: Risk of Progressive Disease According to One Month Post-CAR-T Infusion PET/CT Characteristics for All Patients** | | | |
| --- | --- | --- | --- |
| PET Characteristic | Parameter | Hazard Ratio (95% Confidence Interval) | P-Value |
| Total Metabolic Tumor Volume (MTV) | >55cc | 2.66 (1.32-5.36) | **0.006** |
|  | ≤55cc |  |  |
| Total Lesion Glycolysis (TLG) | >250g | 3.27 (1.40-7.65) | **0.006** |
|  | ≤250g |  |  |
| SUVMax | >10 | 2.75 (1.22-6.22) | **0.015** |
|  | ≤10 |  |  |
| Nodal MTV | >0cc | 1.35 (0.70-2.62) | 0.37 |
|  | 0cc |  |  |
| Bone MTV | >0cc | 1.59 (0.56-4.52) | 0.386 |
|  | 0cc |  |  |
| Parenchymal MTV | >0cc | 2.12 (0.07-4.67) | 0.061 |
|  | 0cc |  |  |
| Soft Tissue MTV | >0cc | 0.92 (0.22-3.82) | 0.904 |
|  | 0cc |  |  |
| MTV of Largest Lesion | >25cc | 2.01 (0.96-4.22) | 0.066 |
|  | ≤25cc |  |  |
| TLG of Largest Lesion | >67g | 1.95 (0.93-4.08) | 0.079 |
|  | ≤67g |  |  |
| Diameter of Largest Lesion | >4cm | 2.41 (1.05-5.52) | **0.038** |
|  | ≤4cm |  |  |
| Number of Discrete Lesions | >2 lesions | 2.56 (1.31-5.00) | **0.006** |
|  | ≤2 lesions |  |  |

| **Supplemental Table 3: One-Month Post-CAR-T Infusion PET/CT Characteristics and Risk of Death** | | | | |
| --- | --- | --- | --- | --- |
| PET Characteristic | Median (Range) | Parameter | Hazard Ratio (95% Confidence Interval) | P-Value |
| Total Metabolic Tumor Volume (MTV) | 5.6 (0.0-1605.2) | >180cc | 4.66 (2.09-10.39) | **<0.001** |
|  |  | ≤180cc |  |  |
| Total Lesion Glycolysis (TLG) | 170.4 (0-13382.0) | >1000g | 2.38 (1.00-5.70 | 0.051 |
|  |  | ≤1000g |  |  |
| SUVMax | 8.7 (3.0-34.8) | >10 | 2.36 (1.01-5.55) | **0.048** |
|  |  | ≤10 |  |  |
| Nodal MTV | 0.0 (0.0-1605.2) | >0cc | 1.42 (0.68-1.96) | 0.351 |
|  |  | 0cc |  |  |
| Bone MTV | 0.0 (0.0-343.0) | >0cc | 2.89 (1.17-7.14) | **0.022** |
|  |  | 0cc |  |  |
| Parenchymal MTV | 0.0 (0.0-62.6) | >0cc | 3.26 (1.50-7.07) | **0.003** |
|  |  | 0cc |  |  |
| Soft Tissue MTV | 0.0 (0.0-60.1) | >0cc | 2.16 (0.65-7.23) | 0.211 |
|  |  | 0cc |  |  |
| MTV of Largest Lesion | 25.1 (0.6-1504.8) | >70cc | 1.99 (0.85-4.66) | 0.113 |
|  |  | ≤70cc |  |  |
| TLG of Largest Lesion | 66.8 (1.9-12312.2) | >245g | 3.70 (1.56-8.81 | **0.003** |
|  |  | ≤245g |  |  |
| Diameter of Largest Lesion | 5.2 (1.4-46.3) | >7cm | 4.22 (1.76-10.08) | **0.001** |
|  |  | ≤7cm |  |  |
| Number of Discrete Lesions | 1.0 (0.0-42.0) | >2 lesions | 11.80 (4.37-31.88) | **<0.001** |
|  |  | ≤2 lesions |  |  |

*Bold font indicates statistical significance*

| **Supplemental Table 4: Association between PET/CT Characteristics and Death in Patients with PR or SD One-Month after CAR-T** | | | |
| --- | --- | --- | --- |
| PET Characteristic | Parameter | Hazard Ratio (95% Confidence Interval) | P-Value |
| Total Metabolic Tumor Volume (MTV) | >150cc | 3.76 (1.11-12.67) | **0.033** |
|  | ≤150cc |  |  |
| Total Lesion Glycolysis (TLG) | >1000g | 4.49 (1.24-16.32) | **0.022** |
|  | ≤1000g |  |  |
| SUVMax | >10 | 3.55 (0.98-12.84) | 0.053 |
|  | ≤10 |  |  |
| Nodal MTV | >0cc | 0.55 (0.16-1.85) | 0.337 |
|  | 0cc |  |  |
| Bone MTV | >0cc | 1.90 (0.24-15.26) | 0.546 |
|  | 0cc |  |  |
| Parenchymal MTV | >0cc | 2.86 (0.90-9.08) | 0.074 |
|  | 0cc |  |  |
| Soft Tissue MTV | >0cc | 2.87 (0.60-13.63) | 0.185 |
|  | 0cc |  |  |
| MTV of Largest Lesion | >150cc | 3.32 (0.85-13.02) | 0.086 |
|  | ≤150cc |  |  |
| TLG of Largest Lesion | >245g | 4.49 (1.24-16.32) | **0.022** |
|  | ≤245g |  |  |
| Diameter of Largest Lesion | >7.5cm | 4.49 (1.24-16.32) | **0.022** |
|  | ≤7.5cm |  |  |
| Number of Discrete Lesions | >2 lesions | 3.56 (0.90-14.03) | 0.07 |
|  | ≤2 lesions |  |  |

*Bold font indicates statistical significance*
